# DNA metabarcoding reveals consumption of diverse community of amphibians by invasive wild pigs (*Sus scrofa*) in the southeastern United States

**DOI:** 10.1038/s41598-023-48139-9

**Published:** 2023-11-28

**Authors:** Vienna R. Canright, Antoinette J. Piaggio, Sarah M. Chinn, Rachael M. Giglio, Joseph M. Craine, James C. Beasley

**Affiliations:** 1 Savannah River Ecology Laboratory, Warnell School of Forestry and Natural Resources, University of Georgia, Aiken, SC USA; 2 U.S. Department of Agriculture, Animal and Plant Health Inspection Service, Wildlife Services, National Wildlife Research Center, Fort Collins, CO USA; 3 U.S. Fish and Wildlife Service, Anchorage, AK USA; 4 Jonah Ventures, LLC, Boulder, CO USA

**Keywords:** Ecology, Invasive species

## Abstract

Invasive wild pigs (*Sus scrofa*) are one of the most widespread, destructive vertebrate species globally. Their success can largely be attributed to their generalist diets, which are dominated by plant material but also include diverse animal taxa. Wild pigs are demonstrated nest predators of ground-nesting birds and reptiles, and likely pose a threat to amphibians given their extensive overlap in wetland use. DNA metabarcoding of fecal samples from 222 adult wild pigs culled monthly from 2017 to 2018 revealed a diverse diet dominated by plant material, with 166 plant genera from 56 families and 18 vertebrate species identified. Diet composition varied seasonally with availability for plants and was consistent between sexes. Amphibians were the most frequent vertebrate group consumed and represented the majority of vertebrate species detected, suggesting amphibians are potentially vulnerable to predation by wild pigs in our study region. Mammal, reptile, and bird species were also detected in pig diets, but infrequently. Our results highlight the need for research on the impacts of wild pigs on amphibians to better inform management and conservation of imperiled species.

## Introduction

Invasive species present a significant threat to global biodiversity and community function that is second only to threats from habitat loss and fragmentation^1^. Invasive wild pigs (*Sus scrofa*), which include Eurasian wild boar outside of their native range, feral domestic pigs, and their hybrids^2,3^, are one of the most widespread and prolific invasive vertebrates globally, occurring on all continents except Antarctica as well as many islands^1,4^. Although the full extent of wild pig impacts on a global scale remains unknown, wild pigs pose a significant threat to hundreds of taxa and have been a primary factor in the extinction of several species^5^. In the United States (U.S.), wild pigs are responsible for a wide range of negative impacts including damage to crops, livestock depredation, disease transmission, destruction of property and ecosystems, and depredation of wildlife^6–9^. Their distributions in the US have been estimated to overlap with over 85% of imperiled species that could be directly impacted by wild pigs through habitat destruction or predation^8^. A growing body of literature^10^ seeks to describe and quantify their economic impact, including to agriculture and natural resources^11,12^.

Wild pigs are dietary and habitat generalists that display a wide array of feeding behaviors, including browsing, grazing, rooting, scavenging, and predation^13–15^. In particular, rooting, where pigs overturn soil in search of food, has been associated with negative impacts to both plants and animals^1,11^. Disturbances from rooting reduce populations and overall diversity of native plant species and allow for the establishment of exotic plants^11,16^. Rooting by wild pigs was also found to disrupt vital montane seep habitat for salamanders, resulting in decreased salamander abundance^17^ and has been implicated in the declines of Southern Dusky Salamanders (*Desmognathus auriculatus*)^18,19^. Garabedian et al.^20^ found that white-tailed deer (*Odocoileus virginianus*) alter their fine scale movements and space use in response to presence of even low densities of invasive wild pigs, suggesting an attempt at reducing competition. A broad range of taxa are thus impacted by wild pigs, with these impacts reaching from individual to community levels of organization.

In addition to indirect disruptions caused by rooting behaviors of wild pigs and wild boar, their generalist diets allow them to consume a wide variety of taxa, creating direct impacts on species through predation^21–24^. Plants make up the largest component of diets of both wild pigs and wild boar in their native range, constituting 62–100% by volume and occurring in nearly 100% of stomach samples^21–23,25^. Wild pigs have also been found to consume fungi, insects, mollusks, crustaceans, fish, amphibians, reptiles, birds, and mammals^25^. Earthworms are commonly consumed and often the most frequent animal material detected^23,25–27^. Among vertebrate groups consumed, small mammals such as California voles (*Microtus californicus*) and Botta’s pocket gophers (*Thomomys bottae*) have been found to occur in wild pig diets at high frequencies, with evidence of targeted predation (e.g., Refs.^1,2^). While predation has been documented, Wilcox and Van Vuren^14^ noted that the vertebrates consumed were primarily fossorial or semi-fossorial small mammals and thus could have been taken opportunistically.

Although studies are limited, amphibians, reptiles, and ground-nesting birds could be similarly at risk of predation by wild pigs and native wild boar^14,24,26,28,29^. Within their native range, wild boar consume chicks and eggs of ground-nesting birds, making them a potential threat to the conservation of these species^29–31^. Invasive wild pigs have also been found to impact native birds. For example, on a small Australian island, wild pigs were implicated in the decline of a flightless bird, the Lord Howe Island woodhen (*Gallirallus sylvestris*), which was able to expand its distribution on the island following removal of wild pigs^32^. While amphibians and reptiles have thus far been found to occur at low frequencies in wild pig diets, Risch et al.^5^ described herpetofauna as the taxa proportionally most threatened by wild pigs in Australia, the U.S., and Europe based on the International Union for the Conservation of Nature’s (IUCN) Red List of Threatened Species. This could largely be due to the overlap in use of wetlands between wild pigs and amphibians. Amphibians are known to concentrate in wetlands during breeding season and typically remain within a kilometer of breeding habitat the rest of the year^33^, and wild pigs select for wetlands and other habitats in proximity to water^34^. The significant threats posed by invasive wild pigs creates an urgent need for understanding the extent of these impacts, including wild pig predation on native vertebrate species.

The generalist and omnivore diet of wild pigs and native wild boars allows them to alter their diets as needed across locations and seasons depending on availability^13,24,35,36^. For example, while plants dominate the diets of *Sus scrofa* overall, wild pigs consume a higher proportion of animal matter and fungi than wild boar in their native range^25^. Seasonal variability in wild pig diets has primarily been attributed to plant availability. For example, acorns are a prominent component of the diets of both wild pigs and wild boar during fall and winter^13,35^. Rooting for underground food items was also observed during winter, when above-ground vegetation was limited^37^, while herbage and foliage increased in importance during the spring growing season^38^. Studies describing seasonal trends in the consumption of animals are more limited but provide further evidence of opportunistic feeding. For instance, wild boar consumed ducks more frequently during molting season when they were more vulnerable to predation^35^. Jolley et al.^28^ detected green anoles (*Anolis carolinensis*) and eastern fence lizards (*Sceloporus undulatus*) in December and January, when the lizards and anoles were likely most available to wild pigs as they sought warmth in leaf litter. Due to this extensive seasonal variability, it is important to conduct year-round investigations of wild pig diets to create a full picture of their potential impacts to native species.

Although studies are more limited, sex is another potential factor influencing food selection by wild pigs, as females have the added energetic cost of reproduction and caring for large litters of young, sometimes multiple times per year^39^. Most studies have not found evidence of differences in diet composition between males and females^13,40,41^. However, Wilcox and Van Vuren^14^ found that female wild pigs appeared to consume higher frequencies of vertebrates during summer and fall compared to males, which corresponded to periods of reduced physical condition (measured by rump fat) in their sampled wild pigs. They suggested that females might increase consumption of protein-rich vertebrate species due to higher energetic costs of reproduction^14^. These conflicting results suggest that more research is needed to investigate the potential effect of sex on wild pig food selection throughout the year.

Most wild pig and native wild boar diet studies to date have relied on visual examination of stomach contents (e.g., Refs.^22,35,42^). However, omnivorous diets present a unique challenge for visually examining stomach contents, as food materials can have differential rates of digestion, with difficult-to-digest plant materials remaining easier to detect and identify than animal materials such as egg shells and soft tissues, which quickly degrade and can be underrepresented^24,30,43^. Molecular methods such as DNA metabarcoding can provide a more sensitive and comprehensive analysis of diet from fecal samples^44^, and are increasingly being used to characterize omnivore diets^43^. However, use of molecular methods to assess wild pig and wild boar diets remains limited^23,24,30,45^. Due to the concern regarding predation of vertebrates, more diet studies using DNA metabarcoding are needed to ensure predation events of vertebrate taxa are not underestimated. In particular, the southeastern U.S. was identified as a region of biodiversity conservation concern^46^ and accounts for over half of herpetofauna species diversity in Canada and the U.S., as well as many mammal and bird species^47–49^. However, wild pig dietary studies within this region are limited^23,24,38^, despite long-established wild pig populations^50,51^.

The goal of this study was to use DNA metabarcoding to assess the potential impacts of invasive wild pig diet on native plant and vertebrate species in South Carolina, U.S. Our objectives were to (1) characterize the vegetation and vertebrate communities consumed by wild pigs in South Carolina, (2) quantify differences in the dietary beta diversity of wild pigs across seasons and between males and females, and (3) identify which taxa appear most susceptible to predation by wild pigs in this study region. We predicted that diet composition would vary seasonally with availability. For example, oak (*Quercus* spp.) would be consumed most frequently in fall and winter months when acorns were available in higher quantities. Additionally, we hypothesized that consumption of plant material would be the same between males and females, but that vertebrates would be consumed more by females than males. Finally, we hypothesized that more amphibians would be detected in wild pig diets compared to previous studies due to the high amphibian diversity and abundance in the region, the shared use of habitats surrounding wetlands by amphibians and wild pigs, and our use of DNA metabarcoding as opposed to traditional dietary methods..

## Results

### Diet overview

Of the 222 samples collected, two were removed prior to analysis as they did not meet the criteria of taxonomic resolution down to family level. The final data set used for statistical analysis represented approximately balanced sexes, with 116 female samples, 101 male samples, and three of unknown sex. Across all samples, the total number of reads was 3,137,261 for plant (trnL) results and 8763 for vertebrates (12S rDNA), with a mean read count of 14,260 per sample for plant data and 39.8 per sample for vertebrate taxa. The mean number of plant families consumed by individual wild pigs was 8 (± 3, SD) and mean number of vertebrate species detected per sample was 0.11 (± 0.50, SD). Only 17 samples out of the original 222 contained vertebrate prey items and of these, 12 were female and 5 were male wild pigs.

**Figure 1:**
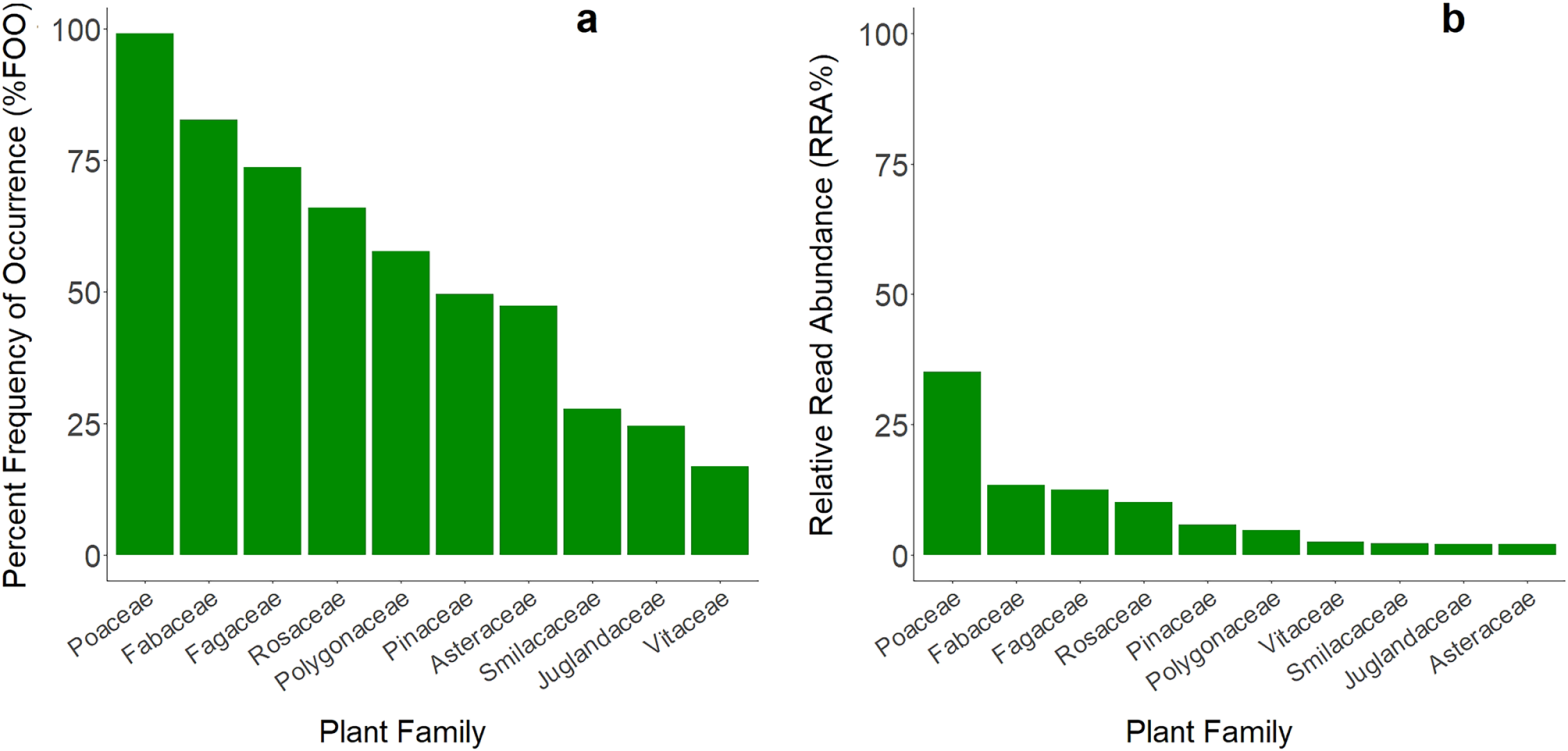
(**a**) Percent frequency of occurrence (%FOO; number of samples containing each food taxa divided by total number of samples and expressed as a percentage) of the 10 plant (trnL) families occurring most frequently throughout the year overall in the diet of wild pigs (*Sus scrofa*) in South Carolina, U.S. June 2017–September 2018; and (**b**) Relative Read Abundance (RRA%; total number of reads of each plant family divided by total number of reads and expressed as a percentage) of the top 10 plant (trnL) families most abundant throughout the year overall in the diet of our sampled wild pigs.

Plants were consumed more frequently than vertebrates, occurring in 100% of samples (Supplementary Table S1). Across all samples, 166 plant genera belonging to 56 plant families were recorded. The most frequent plant families also had the highest RRA in the diet, although the rank order of some families was slightly different between the two metrics (Fig. 1). Poaceae (grasses), Fabaceae (legumes; e.g., *Apios* spp., *Desmodium* spp., *Trifolium* spp.), Fagaceae (hard-mast species; i.e., *Quercus* spp.), and Rosaceae (forbs, soft-mast species; e.g., *Potentilla* spp., *Rubus* spp., *Prunus* spp.) were both the most frequently occurring and most abundant plant families, in descending order.

**Figure 2:**
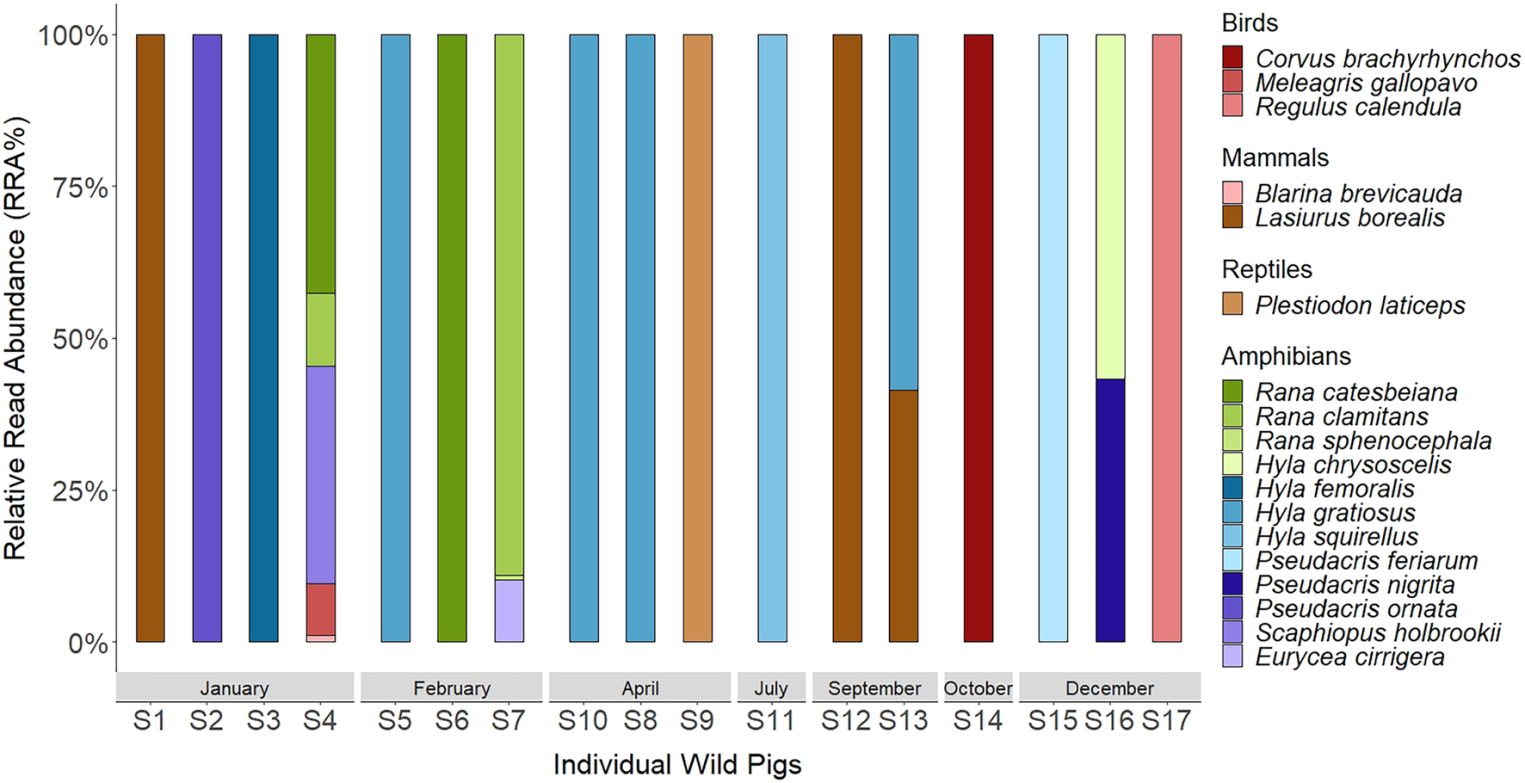
Relative Read Abundance (RRA%; number of reads of each vertebrate species divided by total number of vertebrate reads per sample) of vertebrate species (12S mitochondrial rDNA) detected in each wild pig (*Sus scrofa*) diet sample per month in South Carolina, U.S. in June 2017–September 2018.

**Figure 3:**
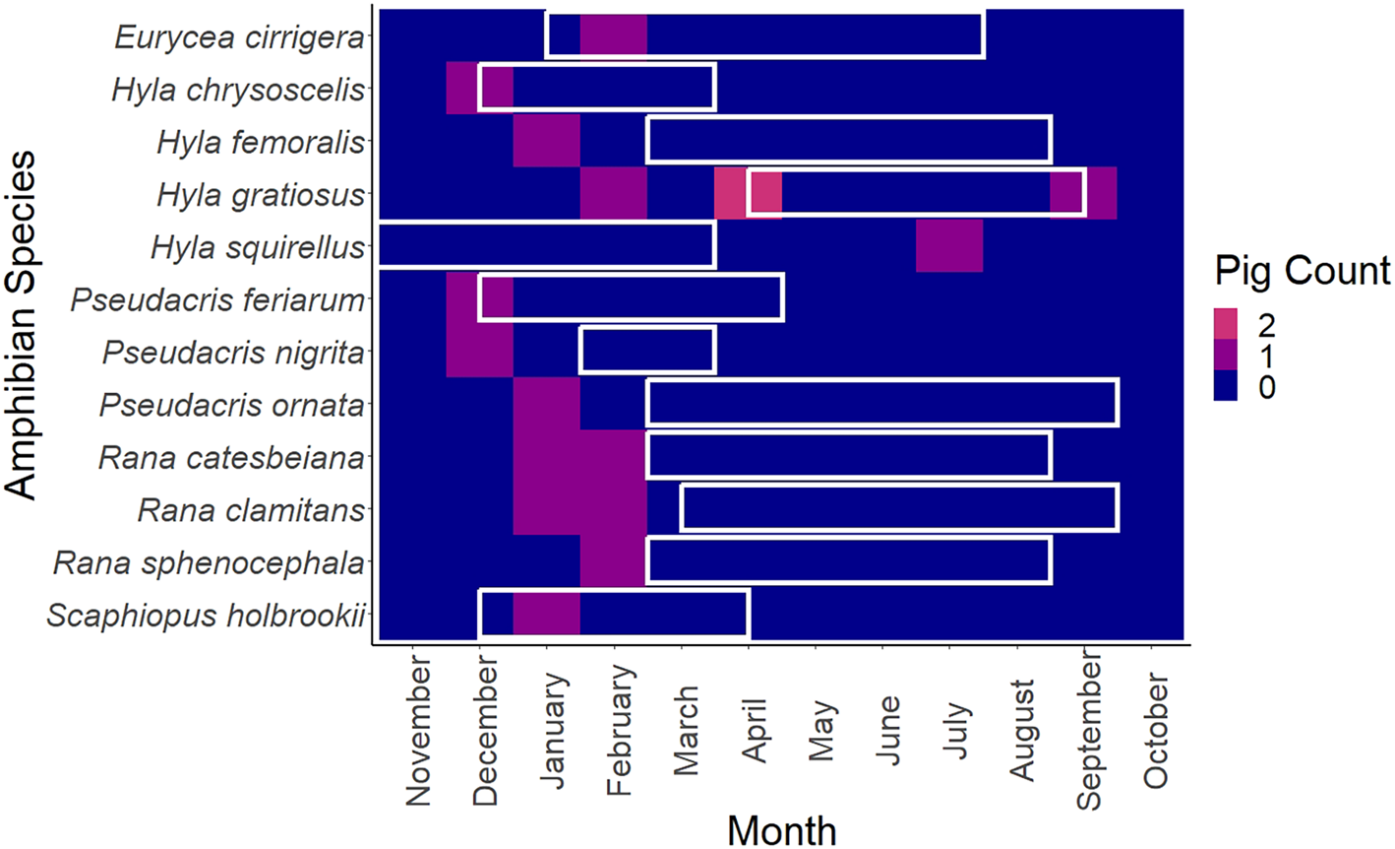
Number of wild pig (*Sus scrofa*) diet samples collected in South Carolina, U.S. in June 2017–September 2018 containing each detected amphibian species (12S mitochondrial rDNA) per month. White boxes indicate approximate breeding season of each amphibian species.

Vertebrates were identified to 18 species belonging to four classes (Amphibia, Reptilia, Mammalia, and Aves) (Table 1). Amphibians were the most frequent vertebrate group detected, occurring in 71% of samples with vertebrate DNA. Amphibians were also the most diverse group of vertebrates consumed, representing 12 of the 18 species detected. We detected more amphibian species in sampled wild pigs than other US studies, including studies in the southeastern region, which identified 0–5 amphibian species in wild pig diets^13,23,28,38^. Barking tree frogs (*Hyla gratiosa*) were the most frequently detected vertebrate, occurring in 4 samples. Mammal, reptile, and bird species were detected infrequently, with eastern red bat (*Lasiurus borealis*) occurring most frequently in 3 samples. American crow (*Corvus brachyrhynchos*), wild turkey (*Meleagris gallopavo*), ruby-crowned kinglet (*Regulus calendula*) were the 3 bird species detected. Two mammal species, short-tailed shrew (*Blarina brevicauda*) and eastern red bat, and 1 reptile species, broadhead skink (*Plestiodon laticeps*), were also detected (Table 1). We observed vertebrates in the diet primarily during late autumn through early spring, with only one sample containing vertebrates between May and August (Fig. 2). Amphibians, the dominant vertebrate taxon sequenced, drove this trend, with 88.2% of amphibian detections occurring December–April. Of the 17 amphibian detections found, 44.2% occurred during the amphibian breeding seasons (Fig. 3)^49^.

**Table 1:** Percent frequency of occurrence (%FOO; number of samples containing each food taxa divided by total number of samples [n = 220] and expressed as a percentage) and Relative Read Abundance (RRA%; total number of reads of each vertebrate species divided by total number of vertebrate reads and expressed as a percentage) of vertebrate species (12S mitochondrial rDNA) detected in the diet of wild pigs (*Sus scrofa*) in South Carolina, U.S. in June 2017–September 2018; “n” represents number of wild pig samples containing that vertebrate species.

Class	Species	Common name	n	FOO %	RRA %
Amphibia	*Hyla chrysoscelis*	Cope's gray treefrog	1	0.45	0.43
	*Hyla femoralis*	Pine woods treefrog	1	0.45	1.77
	*Hyla gratiosus*	Barking treefrog	4	1.82	5.48
	*Hyla squirellus*	Squirrel treefrog	1	0.45	1.88
	*Pseudacris feriarum*	Upland chorus frog	1	0.45	0.24
	*Pseudacris nigrita*	Southern chorus frog	1	0.45	0.33
	*Pseudacris ornata*	Ornate chorus frog	1	0.45	1.31
	*Rana catesbeiana*	American bullfrog	2	0.91	21.92
	*Rana clamitans*	Green frog	2	0.91	23.54
	*Rana sphenocephala*	Southern leopard frog	1	0.45	0.14
	*Scaphiopus holbrookii*	Eastern spadefoot toad	1	0.45	15.70
	*Eurycea cirrigera*	Southern two-lined salamander	1	0.45	2.12
Reptilia	*Plestiodon laticeps*	Broadhead skink	1	0.45	1.81
Aves	*Corvus brachyrhynchos*	American crow	1	0.45	13.57
	*Meleagris gallopavo*	Wild turkey	1	0.45	3.74
	*Regulus calendula*	Ruby-crowned kinglet	1	0.45	3.75
Mammalia	*Blarina brevicauda*	Short-tailed shrew	1	0.45	0.47
	*Lasiurus borealis*	Eastern red bat	3	1.36	1.78

### Beta diversity

**Figure 4:**
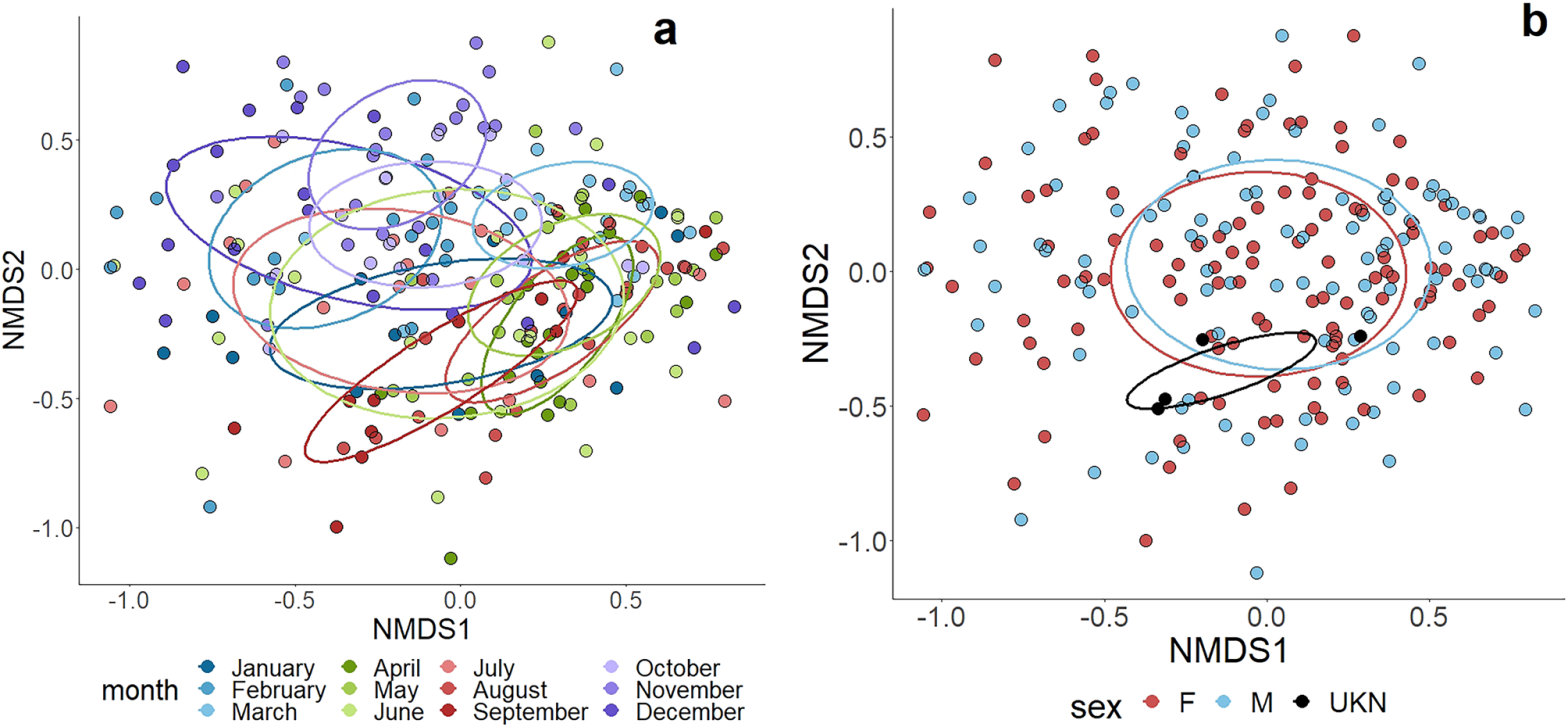
(**a**) Non-metric multidimensional scaling plot for plant families (trnL) detected in the diet of wild pigs (*Sus scrofa*) in South Carolina, U.S. in June 2017–September 2018 by month; and (**b**) non-metric multidimensional scaling plot for plant families (trnL) detected in the diet of wild pigs (*Sus scrofa*) in South Carolina, U.S. in June 2017–September 2018 by sex.

For plant abundance data, the NMDS randomization test converged on a stress value of 0.19, indicating that individual dissimilarities between plant compositions were effectively captured with 3 dimensions. The ANOSIM for plant beta diversity revealed a significant effect of month (p < 0.001, R = 0.21), with fall and winter months clustered distinctly from spring and summer months (Fig. 4). In contrast, sex did not appear to influence plant beta diversity in the diet as neither sex, nor the interaction of month and sex significantly affected plant beta diversity (Fig. 4).

All pairwise comparisons of plant beta diversity among months conducted with a PERMANOVA were significant (FDR adjusted p < 0.05) with the exception of mid-winter months (January x February) and late spring through early fall months (June x May, July x May, June x July, and June x September) (Supplementary Table S2). Diet composition thus varied between most months but did demonstrate some level of seasonality with some months within seasons having similar compositions.

**Figure 5:**
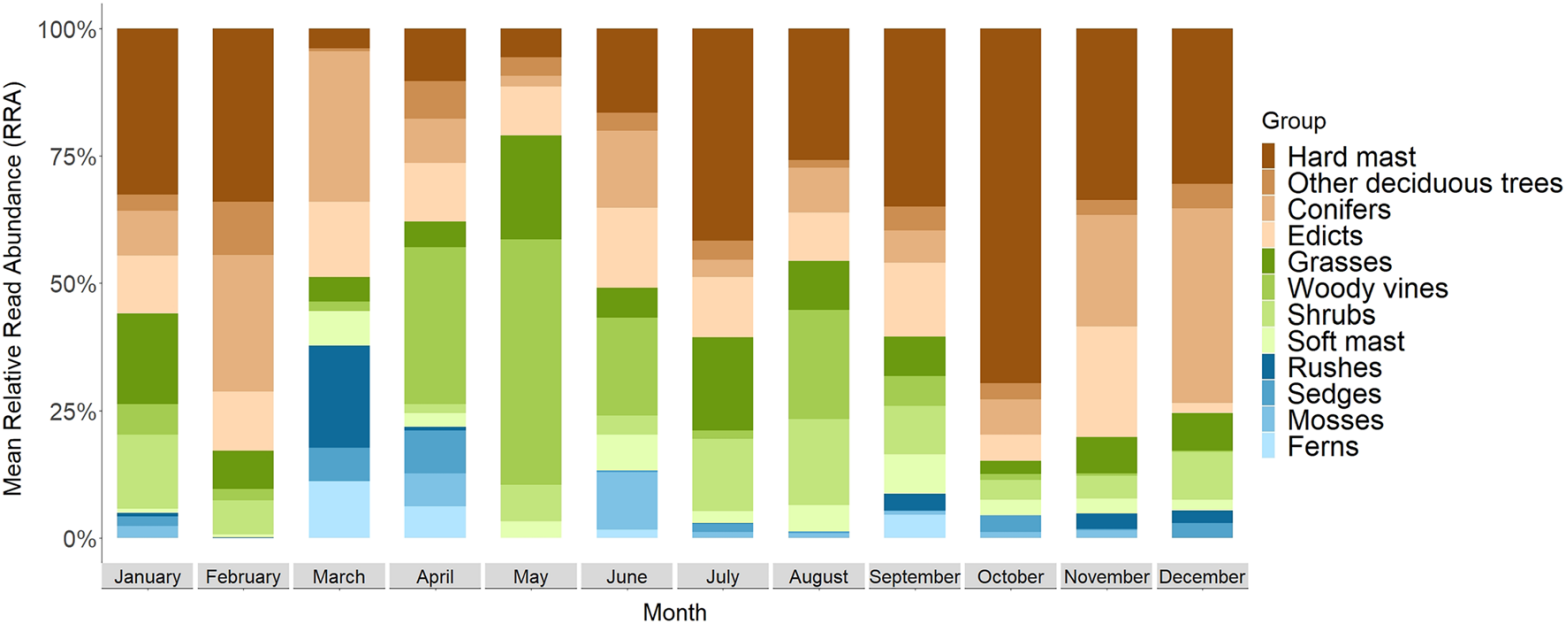
Mean Relative Read Abundance (RRA%; %; mean number of reads of each plant family per month divided by total number of reads per month and expressed as a percentage) of plant functional groups (trnL) detected in the diet of wild pigs (*Sus scrofa*) in the South Carolina, U.S. per month for samples collected in June 2017–September 2018. Assignment of plant genera to groups utilized in Fig. 5 is outlined in Supplementary Table S3.

The Indicator Species Analysis revealed 16 indicator plant families out of 56 families detected (Supplementary Fig. S1). Poaceae and Fabaceae were the most frequently occurring plant families and were selected as indicator families throughout most of the year. Fagaceae (hard-mast) was found to be an indicator family for fall months, as well as July (Fig. 5). Juglandaceae (e.g., *Carya* spp.), also hard-masting species, was also an important dietary component during fall months. Pinaceae (pines) was a significant component of the diet in early fall and spring. In spring and summer months, soft-mast producing plants (Rosaceae) as well as shrubs, edicts, and vines (Amaryllidaceae, Arecaceae, Commelinaceae, Polygonaceae, Salicaceae, Smilacaceae, Violaceae, Vitaceae, and Zygophyllaceae) were identified as indicator families in the diet composition.

**Figure 6:**
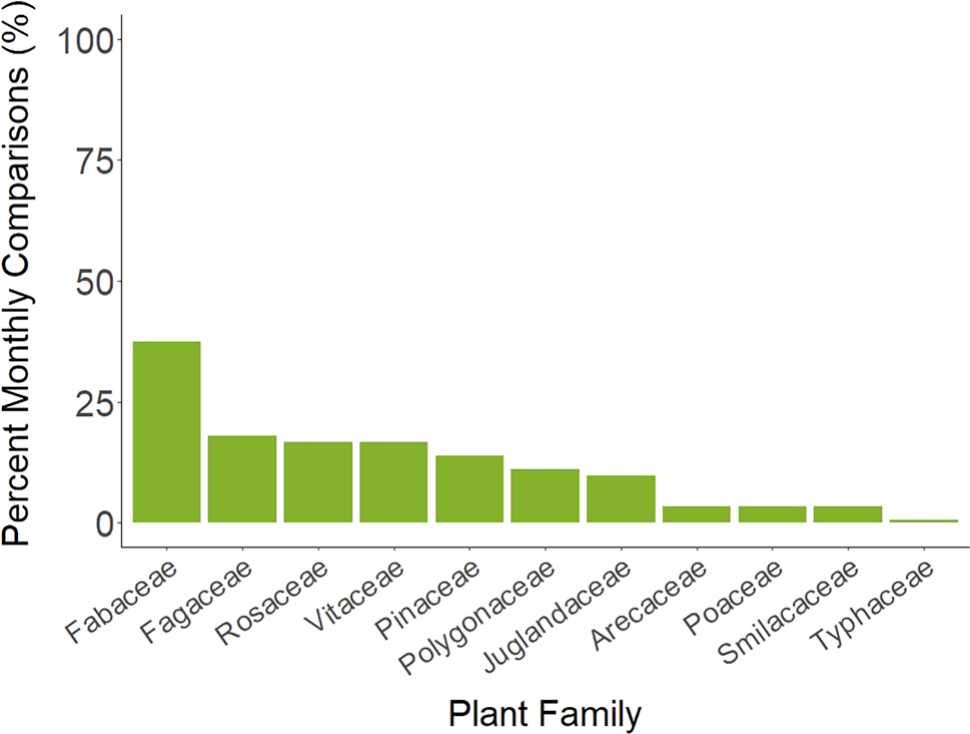
Percent of monthly pairwise comparisons of plant families (trnL) contributing to ≥ 50% of monthly variation in diet composition of wild pigs (*Sus scrofa*) in the South Carolina, U.S. per month for samples collected in June 2017–September 2018 derived from the SIMPER.

The trends of plant dietary composition identified by the SIMPER were similar to those revealed by the Indicator Species Analysis (Supplementary Table S4). Poaceae, Smilacaceae, Fagaceae, Rosaceae, Areacacaea, Fabaceae, and Pinaceae were identified as the plant families contributing to the most dissimilarity between months, followed by Juglandaceae, Vitaceae, Polygonaceae, and Typhaceae, respectively (Fig. 6). At least one of these 11 plant families was found to significantly contribute to dissimilarity between months in 62 comparisons (p-value < 0.05; Supplementary Table S4). We found that Poaceae, Fabaceae, and Rosaceae were also identified as key plant families in majority of our pairwise comparisons (n = 66, 63, and 47 comparisons of 66 respectively).

### Compositional data analysis for plant data

To account for the compositional nature of our data, we transformed the plant data using a centered log-ratio transformation (CLR) and conducted an ANOSIM analysis with this transformed data. Using CLR transformed data did not appear to have differing results from the ANOSIM with raw abundance data. Month significantly influenced the beta diversity of consumed plants (p = 0.001, R = 0.08). Sex and the interaction of sex and month were not significant.

## Discussion

Wild pigs are one of the most invasive species globally, and present a threat to countless species worldwide^5,8^. Examining their diets can provide insight into taxa that might be vulnerable to wild pig consumption and inform management and conservation decisions. Using DNA metabarcoding of wild pig fecal samples collected across a two-year period, our study revealed a highly diverse diet, with 166 plant genera from 56 families and 18 vertebrate species identified. Plants dominated the diet in both abundance and frequency of occurrence, and diet composition varied seasonally but not between sexes. Although vertebrates were consumed at lower frequencies compared to plant matter, we detected vertebrates spanning a relatively wide breadth of taxa, including terrestrial vertebrate groups with fossorial, semi-fossrial, and terrestrial habits thought to be vulnerable to wild pig consumption (amphibians, reptiles, small mammals, and ground-nesting birds). In particular, amphibians appear to be among the vertebrate classes more susceptible to predation by wild pigs within our study region, occurring most frequently and representing the majority of the species detected. Given current global declines in many amphibian populations^52,53^ and extensive overlap in habitat use surrounding wetlands by both wild pigs and amphibians^17,54–56^, our results highlight the potential vulnerability of amphibian populations to predation by wild pigs. Thus, this underlines the need for focused management of invasive pigs in localized habitats containing populations of imperiled species.

Consumption of plant material changed throughout the year with availability, as demonstrated in previous studies of wild pig and native wild boar diets^25^. However, DNA metabarcoding allowed for identification of a greater taxonomic breadth to a finer resolution than many traditional wild pig diet studies^22,38,57^. Grasses were the most common and abundant vegetation consumed throughout the year, which is consistent with other studies in the region^23,38^. Edicts (e.g., Fabaceae) were also observed consistently across seasons. As predicted, fall and early winter months were characterized by hard masting species, with consumption of oaks and hickory peaking in October but continuing through March^13,22,23^. Pines also were dominant in the diet during fall months, although this could have been incidental consumption of pine needles during rooting within pine stands. As hard mast availability dwindled in late winter and early spring, pines remained dominant in the diet along with ferns and wetland species such as *Sparganium* spp. and *Typha* spp., although pine detected during spring could in part be pollen. Corresponding to increased plant growth in spring and summer, wild pig diets increased in herbaceous vegetation including woody vine species (e.g., *Smilax* spp.) and soft-masting species (e.g., Rosaceae). We also observed a notable increase in consumption of oaks in July, likely comprising oak seedlings for which wild pigs are known consumers^58^. This summer spike in consumption of oak indicates that targeted temporal and spatial removal of wild pigs might be needed in areas where oak recruitment is of management concern.

Compared to plants, DNA metabarcoding performed better for vertebrate taxa, allowing us to identify all detected vertebrates to species level. Although vertebrates were consumed less frequently than plants, wild pigs consumed species belonging to all four vertebrate groups of interest (amphibians, reptiles, small mammals, and ground nesting birds). It is important to note that absence of earthworms and other invertebrates in this study was due to our decision to focus on vertebrates with a vertebrate-specific primer, not necessarily lack of consumption by our sampled wild pigs. Amphibians were the primary class of vertebrates detected, comprising 12 of the 18 vertebrate species. This represents the most amphibian species identified in wild pig diets in the U.S. to date, with prior studies detecting 0–5 amphibian species^13,23,28,38^. It’s possible that other studies using traditional methods might have underestimated amphibian presence due to rapid digestion. Anderson et al.^23^ used metabarcoding to examine wild pig diets in Florida and detected only 5 amphibian species but used a different 12S primer set that was not designed for *Bactrachia* amplification. Of the 12 amphibian species detected in our study, only one salamander, the southern two-lined salamander (*Eurycea cirrigera*) was found. The low numbers of salamander detections were surprising considering the fossorial habits and high abundance of salamanders in our study region^49,59^. For example, we expected to detect Ambystoma spp. in the diets of wild pigs in our study area as these are semi-fossorial and common in and around wetlands. An in silico analyses performed by Jonah Ventures, LLC for our primer set revealed that the Batr01 primer set reference database is biased against some groups of salamanders including Ambystomatidae, suggesting that more salamanders could have been consumed by wild pigs in our study than shown here.

Consumption of amphibians appeared to demonstrate a seasonal trend, with detections clustered between late fall and early spring. Our amphibian detections were both within and outside the known breeding seasons of these amphibians^49^. DNA metabarcoding does not enable us to determine whether the amphibians detected in pig diets were eggs, larvae, or adults, or if individuals were deceased prior to consumption so we are limited in our current understanding of when and which habitats amphibians are most vulnerable. Given the rooting habits of pigs, we expect they are most likely consuming adults or juveniles around and within wetlands. Mortality to adults and juveniles could have more significant impacts on amphibian populations than would scavenging of remnant tadpoles in drying wetlands^60^.

Despite concerns regarding wild pig and wild boar predation on ground-nesting birds and reptiles^12,26,30,32^ we found limited evidence of this occurring among the individuals sampled in our study. Wild turkey (*Meleagris gallopavo*) was a species of interest in our study area as a ground-nesting game bird, but was only detected in a single occurrence, and the timing of this detection in January (outside of nesting season) suggests this was likely a scavenged adult and unrelated to nesting behavior. Furthermore, only one reptile, the broadhead skink (*Plestiodon laticeps*) was detected in a single sample in April. While other studies in the U.S. have seen higher occurrences of small mammals in wild pig diets with over one third of samples containing small mammals^14,23^, the short-tailed shrew (*Blarina brevicauda*) was the only small mammal detected in our study and occurred in only one sample. These findings suggest that in South Carolina, amphibians appear to be among the more vulnerable wild pig prey groups in contrast to other vertebrate taxa that have been documented in the diet at higher levels elsewhere^14,35,61^. However, given the dominance of plants, more in-depth studies focused within periods of peak vulnerability of ground nesting birds or other concerned taxa (e.g., nesting seasons) are needed to fully capture the potential extent of impacts of wild pig predation.

When using molecular methods, primer biases can influence the breadth and depth of species detected^62,63^. While our specific 12S primer^64^ biases could have potentially led to underrepresentation of bird, mammal, and reptile taxa detection in our study, Kluever et al.^65^ detected multiple local bird and mammal species in the diets of coyotes using the same primer set used in our research. This suggests that primer biases likely played a minimal role in our infrequent detections of birds and mammals and that the species we detected among these groups are likely representative of the actual species consumed by the sampled individuals. However, an in silico analysis by Jonah Ventures, LLC determined a bias of our Batr01 primer set against reptiles in addition to salamanders, suggesting that wild pigs could have consumed more reptile species than we were able to identify. Given the presence of small fossorial snakes and lizards on the SRS^66^, reptiles were likely underrepresented in our study.

Surprisingly, eastern red bats were detected more frequently than birds, reptiles, and other mammals. This is the first known documentation of wild pig consumption of a bat species. Eastern red bats are arboreal and select winter roosts in midstory to understory locations and occasionally in the leaf litter when temperatures are 0–10 °C, potentially explaining the detection in January^67^, as an individual could have been accessible to a wild pig during torpor. However, temperatures did not drop below that threshold during our study period for the September detections^67,68^, which could have reflected scavenging of carcasses, predation of recently volant juveniles, or coprophagy of bat guano as DNA metabarcoding does not allow us to differentiate these forms of consumption from predation^24^.

While RRA can be tentatively interpreted as a semi-quantitative estimate of dietary importance, it is not a reliable predictor of number of individuals of each species consumed and thus we cannot determine how many individual vertebrates were consumed in each sample^63^. As opportunistic foragers, wild pigs have been documented to consume large quantities of a single food item within a short period, with one stomach containing as many as 49 eastern spadefoot toads^28^. Therefore, our results are likely a conservative estimate of the number of individual vertebrates actually consumed. Furthermore, we sampled trapped wild pigs that consumed corn at bait sites for several days prior to capture while conditioning to the trap site. As trapped wild pigs thus had some level of a supplemented diet of easily accessible corn, they could have been consuming less vertebrates than wild pigs that were not being trapped and therefore were not provided with any level of diet supplementation, and the extent of vertebrates in wild pig diets may have been underestimated in our study compared to other scenarios.

Our findings suggest that wild pigs have the potential to pose an important predation risk to amphibian populations. As wild pigs prefer wetland habitat and forage within the leaf litter and upper soil layers^34,69^, they are likely to encounter amphibians frequently, particularly in regions of the world with high amphibian diversity, such as the southeastern U.S^49^. Wild pigs are notorious for destroying critical wetland habitats through rooting while foraging^17,55,56,70^, which may further exacerbate their impacts to vulnerable amphibian communities. As primarily opportunistic feeders, wild pigs could consume high volumes of amphibians in a short time period^28^. If they happen to encounter an amphibian breeding event while foraging, this could be detrimental to localized amphibian populations through the additive effect of direct predation and indirect habitat loss, particularly for those species that are already imperiled. While no threatened or endangered species were detected among our samples, this was not surprising due to the inherent low availability of rare species on the landscape. When combined with short duration over which dietary studies reflect consumed food items before they pass through the digestive system and the relatively low frequency that vertebrates were consumed (< 8%) in this study, it is not unlikely that rare species would go undetected. However, we detected fossorial or semi-fossorial species that spend time in leaf litter and near wetlands which are life history traits similar to several species of concern in the southeastern U.S. such as gopher frogs (*Rana capito*) and reticulated flatwoods salamander (*Ambystoma bishopi*). Coupled with previous research on vertebrate species frequently detected in wild pig diets^14,28^, this suggests that species with these life history characteristics have the potential to be vulnerable to depredation by wild pigs in areas where their ranges overlap. Furthermore, our results provide further evidence that wild pig food habits can pose potential threats to imperiled wetland habitats and oak sapling recruitment, and thus would benefit from management of wild pig populations.

Collectively, our findings highlight the need for further research into the extent to which wild pigs may pose a threat to amphibian populations globally, both directly from predation but also indirectly through habitat modification during rooting. Additional spatio-temporal studies using molecular approaches across larger regions within biodiversity hotspots are needed to determine the extent that amphibians, bats, and imperiled species are being consumed across their range. Finally, more extensive targeted sampling in areas with species of concern should be conducted and more common species with similar life history traits could be utilized as a proxy for rare species to provide insight into how to approach management of wild pigs to best reduce the effects of wild pigs on taxa that are most vulnerable to their impacts.

## Methods

### Study area

This study was conducted at the U.S. Department of Energy’s Savannah River Site (SRS) in west-central South Carolina. The SRS is located in the sandhills and the upper-coastal plain ecoregions of South Carolina, and dominated by upland pine forest, bottomland hardwood forest, and riparian habitats^85^. Upland pine habitats (~ 50% of site) are comprised primarily of loblolly pine (*Pinus taeda*), longleaf pine (*P. palustris*), and slash pine (*P. elliottii*). Bottomland hardwoods (~ 25% of site) include *Taxodium* spp., *Liquidambar* spp., *Quercus* spp., and *Nyssa* spp. Upland hardwood forest, including *Carya* spp., *Acer* spp., *Quercus* spp., and shrubby/herbaceous habitat cover an additional 18% of the site^71^. The site hosts a high diversity of vertebrate species, with close to 100 herpetofauna species^72^ as well as many birds^73^, and mammals^74^. While the SRS has a perimeter fence to restrict public access to the site, wild pigs are able to move freely across the boundary, and have been present in the area since before the establishment of the SRS^75^. Despite control efforts, wild pigs are abundant and widely distributed across the landscape^76^.

### Data collection

We sampled both male and female wild pigs between June 2017 and September 2018 that were live-trapped and culled as part of ongoing wild pig management on the SRS and individuals that were live captured and released for other research purposes. Sampled wild pigs received some level of dietary supplementation as traps were baited with corn. Fresh fecal samples were obtained in the field from culled wild pigs during necropsy or while under anesthesia from the distal colon/rectum. We sampled individuals evenly across the primary habitat types of the SRS and during all months throughout the study years, with approximately balanced sampling efforts across months and sexes. We selected wild pigs > 1 year of age and larger than 25 kg to ensure they were large enough to consume vertebrate prey items and to be independently foraging. No wild pigs were euthanized specifically for this research. This study was approved by the University of Georgia Institutional Animal Care and Use Committee. All experimental protocols were conducted in accordance with the Institutional Animal Care and Use Committee under University of Georgia protocols A2015 05-004, A2015 12-017, and A2018 06-024. All methods were performed in accordance with the ARRIVE guidelines.

Samples were placed on ice in the field until they were transferred to an ultra-low temperature freezer (approximately – 70 C) within a few hours. Samples were later shipped on dry ice to Jonah Ventures, LLC (Boulder, Colorado, USA) for molecular analysis of food items.

### Laboratory analyses and data curation

All laboratory analyses were conducted by Jonah Ventures laboratory. To amplify plant taxa, we used a primer set targeting a section of the chloroplast trnL (UAA) intron—g (5’-GGGCAATCCTGAGCCAA-3’) and h (5’-CCATTGAGTCTCTGCACCTATC-3’ (Taberlet et al. 2007). To amplify vertebrate taxa, we utilized the Batr01 primer set, which targets the 12S mitochondrial rDNA gene -F (5’-ACACCGCCCGTCACCCT-3’) and R (5’- GTAYACTTACCATGTTACGACTT-3’)^64^ focused on the taxonomic group Batrachia but amplifies other vertebrate groups as well. We selected a vertebrate primer set to exclude invertebrates to prevent the anticipated high volumes of earthworms from masking the targeted but typically less frequent vertebrate taxa that were more central to our research question. Our methods used for DNA isolation and processing of sequences were similar to those described by Robeson et al.^24^ for trnL analyses with an updated form of the UNOISE (v3) pipeline to generate Operational Taxonomic Unit (OTU) sequences as Exact Sequence Variants (ESVs)^77^. Sequences were downloaded from GenBank and top hits with alignment query coverages of at least 90% and identities greater or equal to 85% were selected using NCBI BLAST. This was followed by a custom processing pipeline created by Jonah Ventures. We discarded sequences that could not be identified to the family level or were considered possible contaminants.

### Statistical analysis

All analyses were conducted using R v 4.1.1^78^. Because distinct primers were used for plant and vertebrate data, they were examined separately. All analyses were performed on read count (abundance) data at the family level of taxonomic resolution unless otherwise stated. We also calculated both percent Frequency of Occurrence (%FOO) and Relative Read Abundance (RRA)^63^ for use in visualizations and semi-quantification of the diet. %FOO is considered a more conservative approach to interpreting diet data, but it can lead to overestimation of low abundance food items, since as an occurrence metric all food items are given the same weight. RRA, or relative abundance, eliminates this concern but can be influenced by recovery biases, and is thus not always an accurate representation of the relative abundance of the food that was actually consumed^63^. To account for these concerns and for generalizability, we provided both. We calculated %FOO for each food item by dividing the number of samples containing that food by the total number of samples, multiplied by 100. RRA was calculated by dividing the read count of each food item by the total number of reads for that marker and expressed as a percent.

For beta diversity, or diet composition, our data violated assumptions of normality, and we thus conducted non-metric multidimensional scaling (NMDS) to visualize trends between months and sexes. A three-dimensional solution from the lowest stress was used to run a randomization test with 1000 permutations. We then utilized a non-parametric Analysis of Similarities (ANOSIM) with Bray–Curtis distance with 9999 permutations to determine the effects of sex and month on beta diversity of wild pig diets. For pairwise comparisons of plant dietary beta diversity between months we conducted a PERMANOVA with Bray–Curtis distance with 9999 permutations and False Discovery Rate (FDR) corrected p-values. The NMDS, ANOSIM, and PERMANOVA were conducted using the vegan package in R^79^. Alpha diversity was also calculated and descriptions of these methods and results are provided in the Supplementary Information.

To further explore trends of plant beta diversity, we conducted a Similarity Percentages (SIMPER) analysis with 999 permutations using the vegan package in R^79^, and identified the plant families contributing to at least 70% of differences between months. Additionally, we conducted an Indicator Species Analysis using the indicspecies package in R to further examine effects of month on beta diversity^80^. In this analysis, an Indicator Value index is assigned to examine the relationship between each species (or taxon) within a community and the site group (or month). Permutations are used to identify statistically significant taxa that are most representative of the community at the given location or time, based on abundance and occurrence^81^. We used this analysis to identify the significant indicator species (or taxa) for each month. Taxa with higher Indicator Values are more representative of the community during that sampled month, providing a method to quantify the seasonal trends identified by the ANOSIM.

For analyses on vertebrates consumed, only 17 samples contained vertebrates and thus our analyses had limited power. We have therefore only included descriptive results for vertebrate data. To investigate potential drivers behind trends observed in timing of amphibian detections in the diet, we used estimated breeding seasons of the detected amphibians provided by Jenson et al.^49^.

Finally, due to rising concerns of how the inherent compositional nature of data generated by high-throughput sequencing (HTS) might impact analyses and interpretation of metabarcoding data^82,83^, we conducted compositional data analyses on plant data to ensure our results obtained from traditional statistical methods that ignore the compositional nature of HTS data was not impacting our results^82,84^. Compositional data analyses entail performing ratio transformations to the raw abundance (read count) data and using alternate distance metrics to accommodate for the compositional nature of the data. We conducted the NMDS and ANOSIM again as described above, but replacing raw read count data with centered-log ratio (CLR) transformed data and Bray–Curtis distance with Aitchison distance, the Euclidean distance between CLR data^82,83^.

### Supplementary Information

Supplementary Information.

## Data Availability

The data generated or analyzed during the current study are available from the corresponding author upon reasonable request.
