# Supplementary material for: DNA metabarcoding reveals consumption of diverse community of amphibians by invasive wild pigs (Sus scrofa) in the southeastern United States
Source: Sci Rep. 2023 Nov 28;13:20889. doi: 10.1038/s41598-023-48139-9 (PMC10684498; doi:10.1038/s41598-023-48139-9)
Supplement: Supplementary file 1 — Supplementary Information. [file 41598_2023_48139_MOESM1_ESM.pdf]

DNA metabarcoding reveals consumption of diverse community of amphibians by invasive wild pigs (*Sus scrofa*) in the southeastern United States

Vienna R. Canright[1]*, Antoinette J. Piaggio [2], Sarah M. Chinn[1,3], Rachael M. Giglio[2], Joseph M. Craine[4], James C. Beasley[1]

[1] Savannah River Ecology Laboratory, Warnell School of Forestry and Natural Resources, University of Georgia, Aiken, SC, USA

[2] U.S. Department of Agriculture, Animal and Plant Health Inspection Service, Wildlife Services, National Wildlife Research Center, Fort Collins, CO, USA

[3] U.S. Fish and Wildlife Service, Anchorage, AK, USA

[4] Jonah Ventures, LLC, Boulder, CO, USA

* Corresponding author: e-mail: Vienna.Canright@uga.edu

Supplementary Information

Alpha Diversity

Methods

To examine dietary alpha diversity, we calculated Shannon's Diversity Index using the "diversity" function in the vegan package in R [1]. We tested the data for normality and then conducted an Analysis of Variance (ANOVA) to investigate the effects of month and sex on alpha diversity. This was followed by a Tukey's test using the agricolae package in R in the event that significance ($\alpha = 0.05$) was found [2].

Results

Plant family data had a mean Shannon's diversity index score of $H = 1.24$. An ANOVA of Shannon's diversity index revealed a significant effect of month ($F_{11,208} = 3.13$, $p < 0.01$). Tukey's Honestly Significant Difference (HSD) Test for multiple comparisons for month found that the mean H

score was significantly lower in May (H=1.02) than January (H=1.49; p = 0.02, 95% Credible Interval [C.I.] = 0.03-0.89) and February (H=1.44; p = 0.02, 95% C.I. = 0.03-0.80) but not between other months. Neither sex, nor the interaction of month and sex were significant.

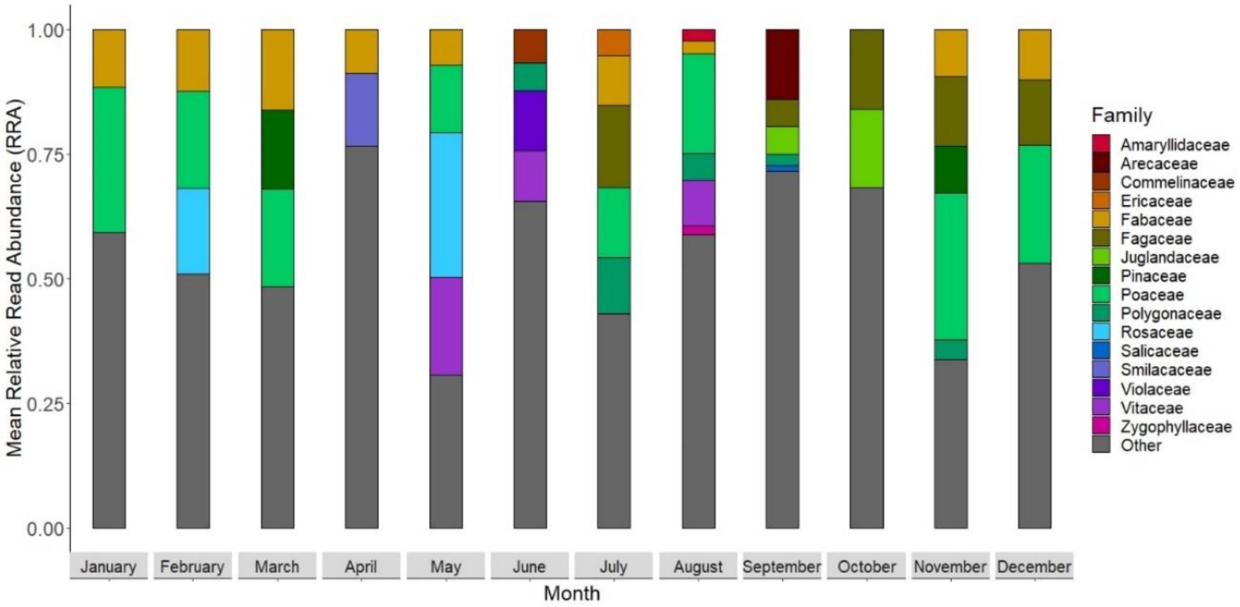

Fig. S1 Mean relative read abundance (RRA) per month of the 16 plant families identified in the Indicator Species Analysis for the diet of wild pigs (*Sus scrofa*) in South Carolina, U.S., June 2017 - September 2018. “Other” represents the RRA of the remaining 40 plant families consumed by wild pigs that did not contribute significantly to variation between months.

Table S1 Number of diet samples containing each food taxa (n), percent frequency of occurrence (%FOO; n divided by total number of samples [220]) and mean relative read abundance (RRA%; number of reads of each food taxa divided by total number of trnL reads) of the plant families detected in the diet of wild pigs (*Sus scrofa*) in South Carolina, U.S.

Family	n	%FOO	%RRA
**Poaceae**	218	99.09	34.56
**Fabaceae**	182	82.73	12.68
**Fagaceae**	162	73.64	13.68
**Rosaceae**	145	65.91	9.72
**Polygonaceae**	127	57.73	4.36
**Pinaceae**	109	49.55	6.12

**Asteraceae**	104	47.27	1.91
**Smilacaceae**	61	27.73	1.81
**Juglandaceae**	54	24.55	3.02
**Vitaceae**	37	16.82	2.66
**Solanaceae**	36	16.36	0.18
**Onagraceae**	30	13.64	0.39
**Cupressaceae**	28	12.73	0.40
**Salicaceae**	27	12.27	0.32
**Arecaceae**	26	11.82	2.45
**Rubiaceae**	26	11.82	0.28
**Altingiaceae**	25	11.36	0.25
**Typhaceae**	23	10.45	1.22
**Caryophyllaceae**	22	10.00	0.14
**Cyperaceae**	22	10.00	0.26
**Plantaginaceae**	22	10.00	0.07
**Chenopodiaceae**	19	8.64	0.17
**Malvaceae**	16	7.27	0.12
**Commelinaceae**	15	6.82	0.24
**Brassicaceae**	14	6.36	0.03
**Geraniaceae**	14	6.36	0.03
**Amaranthaceae**	13	5.91	0.18
**Violaceae**	13	5.91	0.25
**Oxalidaceae**	12	5.45	0.05
**Comandraceae**	11	5.00	0.09
**Juncaceae**	11	5.00	0.22
**Magnoliaceae**	11	5.00	0.15
**Ulmaceae**	11	5.00	0.17
**Anacardiaceae**	9	4.09	0.08
**Euphorbiaceae**	9	4.09	0.04
**Rhamnaceae**	9	4.09	0.13
**Grossulariaceae**	8	3.64	0.04
**Sapindaceae**	8	3.64	0.08
**Araceae**	7	3.18	0.06
**Convolvulaceae**	7	3.18	0.07
**Ericaceae**	7	3.18	0.17
**Cucurbitaceae**	6	2.73	0.05
**Ebenaceae**	6	2.73	0.08
**Amaryllidaceae**	5	2.27	0.07
**Aquifoliaceae**	4	1.82	0.04
**Ditrichaceae**	4	1.82	0.06
**Ephedraceae**	4	1.82	0.06
**Ophioglossaceae**	4	1.82	0.09
**Sphagnaceae**	4	1.82	0.04
**Zygophyllaceae**	3	1.36	0.04

**Berberidaceae**	2	0.91	0.08
**Cabombaceae**	2	0.91	0.29
**Cannabaceae**	2	0.91	0.05
**Polemoniaceae**	2	0.91	0.07
**Rutaceae**	2	0.91	0.04
**Nymphaeaceae**	1	0.45	0.08

Table S2 Results of PERMANOVA of monthly pairwise comparisons for plant families detected in the diet of wild pigs (*Sus scrofa*) in South Carolina, U.S.

**Month 1**	**Month 2**	**R^2**	**FDR adjust P**
December	January	0.0938	0.0012
December	February	0.1560	0.0006
December	March	0.1722	0.0003
December	April	0.1983	0.0003
December	May	0.1962	0.0004
December	June	0.1311	0.0003
December	July	0.1463	0.0003
December	August	0.1434	0.0003
December	September	0.1115	0.0003
December	October	0.1430	0.0003
December	November	0.1019	0.0025
January	December	0.0938	0.0019
January	February	0.0530	0.0663
January	March	0.1304	0.0003
January	April	0.1668	0.0003
January	May	0.0845	0.0285
January	June	0.0989	0.0011
January	July	0.0692	0.0073
January	August	0.1082	0.0003
January	September	0.1115	0.0004
January	October	0.1479	0.0003
January	November	0.0935	0.0037
February	December	0.1560	0.0003
February	January	0.0530	0.0607
February	March	0.0991	0.0016
February	April	0.1541	0.0003
February	May	0.0423	0.2483
February	June	0.1125	0.0009
February	July	0.0732	0.0090
February	August	0.1623	0.0003
February	September	0.1026	0.0008

February	October	0.1871	0.0003
February	November	0.1095	0.0028
March	December	0.1722	0.0003
March	January	0.1304	0.0003
March	February	0.0991	0.0023
March	April	0.1389	0.0003
March	May	0.1144	0.0030
March	June	0.1339	0.0003
March	July	0.1102	0.0004
March	August	0.1905	0.0003
March	September	0.1382	0.0003
March	October	0.2575	0.0003
March	November	0.1243	0.0006
April	December	0.1983	0.0003
April	January	0.1668	0.0003
April	February	0.1541	0.0004
April	March	0.1389	0.0003
April	May	0.1060	0.0228
April	June	0.0767	0.0174
April	July	0.0941	0.0016
April	August	0.1785	0.0003
April	September	0.0974	0.0008
April	October	0.2254	0.0003
April	November	0.1801	0.0004
May	December	0.1962	0.0003
May	January	0.0845	0.0286
May	February	0.0423	0.2483
May	March	0.1144	0.0029
May	April	0.1060	0.0214
May	June	0.0599	0.1086
May	July	0.0365	0.3611
May	August	0.1179	0.0029
May	September	0.0732	0.0250
May	October	0.1915	0.0003
May	November	0.1517	0.0015
June	December	0.1311	0.0003
June	January	0.0989	0.0006
June	February	0.1125	0.0008
June	March	0.1339	0.0003
June	April	0.0767	0.0170
June	May	0.0599	0.1086
June	July	0.0349	0.2483
June	August	0.0642	0.0228
June	September	0.0380	0.1785

June	October	0.1307	0.0003
June	November	0.1005	0.0021
July	December	0.1463	0.0003
July	January	0.0692	0.0069
July	February	0.0732	0.0104
July	March	0.1102	0.0003
July	April	0.0941	0.0016
July	May	0.0365	0.3646
July	June	0.0349	0.2483
July	August	0.0877	0.0008
July	September	0.0501	0.0427
July	October	0.1548	0.0003
July	November	0.1059	0.0004
August	December	0.1434	0.0003
August	January	0.1082	0.0003
August	February	0.1623	0.0003
August	March	0.1905	0.0003
August	April	0.1785	0.0003
August	May	0.1179	0.0022
August	June	0.0642	0.0245
August	July	0.0877	0.0012
August	September	0.0654	0.0136
August	October	0.0924	0.0004
August	November	0.0712	0.0136
September	December	0.1115	0.0003
September	January	0.1115	0.0004
September	February	0.1026	0.0006
September	March	0.1382	0.0003
September	April	0.0974	0.0009
September	May	0.0732	0.0257
September	June	0.0380	0.1785
September	July	0.0501	0.0390
September	August	0.0654	0.0118
September	October	0.0766	0.0036
September	November	0.0767	0.0068
October	December	0.1430	0.0003
October	January	0.1479	0.0003
October	February	0.1871	0.0003
October	March	0.2575	0.0003
October	April	0.2254	0.0003
October	May	0.1915	0.0003
October	June	0.1307	0.0003
October	July	0.1548	0.0003
October	August	0.0924	0.0011

October	September	0.0766	0.0023
October	November	0.0595	0.0370
November	December	0.1019	0.0014
November	January	0.0935	0.0025
November	February	0.1095	0.0034
November	March	0.1243	0.0003
November	April	0.1801	0.0003
November	May	0.1517	0.0018
November	June	0.1005	0.0025
November	July	0.1059	0.0008
November	August	0.0712	0.0138
November	September	0.0767	0.0073
November	October	0.0595	0.0361

Table S3 Breakdown of plant genera and associated family assigned to each plant group in Fig. 5 detected in the diet of wild pigs (*Sus scrofa*) in South Carolina, U.S.

**Plant Group**	**Family**	**Genus**
**Conifers**	Cupressaceae	*Cunninghamia*
	Pinaceae	*Abies*
		Cedrus
		Pinus
		Pseudotsuga
**Deciduous trees**	Altingiaceae	*Liquidambar*
	Betulaceae	*Alnus*
		Betula
	Ebenaceae	*Diospyros*
	Fabaceae	*Robinia*
	Platanaceae	*Platanus*
	Rhamnaceae	*Alphitonia*
	Salicaceae	*Populus*
		Salix
	Sapindaceae	*Acer*
	Simaroubaceae	*Ailanthus*
	Ulmaceae	*Ulmus*
**Ferns**	Ophioglossaceae	*Botrychium*
		Ophioglossum

**Edicts**	Amaranthaceae	*Alternanthera*
		Amaranthus
		Bassia
		Chenopodium
		Oxybasis
	Amaryllidaceae	*Allium*
	Apocynaceae	*Apocynum*
	Araceae	*Steudnera*
	Araliaceae	*Hydrocotyle*
	Asteraceae	*Bidens*
		Lactuca
		Taraxacum
		Tragopogon
	Boraginaceae	*Phacelia*
	Brassicaceae	*Alyssum*
		Brassica
	Caryophyllaceae	*Cerastium*
		Silene
		Stellaria
	Commelinaceae	*Commelina*
	Cucurbitaceae	*Cucumis*
	Euphorbiaceae	*Cnidoscolus*
		Euphorbia
	Fabaceae	*Apios*
		Arachis
		Astragalus
		Dalea
		Desmanthus
		Desmodium
		Glycine
		Hedysarum
		Hoffmannseggia
		Kummerowia
		Lespedeza
		Lotus
		Lupinus
		Medicago
		Melilotus
		Onobrychis
		Pisum
		Rhynchosia
		Trifolium
		Vicia
	Geraniaceae	*Geranium*

	Hypericaceae	*Triadenum*
	Lamiaceae	*Lamium*
	Malvaceae	*Hibiscus*
		Sida
	Onagraceae	*Chamaenerion*
		Ludwigia
		Oenothera
	Oxalidaceae	*Oxalis*
	Passifloraceae	*Passiflora*
	Penstemon	*Penstemon*
	Plantaginaceae	*Linaria*
		Plantago
		Veronica
	Polemoniaceae	*Linanthus*
	Polygalaceae	*Polygala*
	Polygonaceae	*Eriogonum*
		Persicaria
		Polygonum
		Rumex
	Primulcaeae	*Douglasia*
	Rosaceae	*Geum*
		Potentilla
	Rubiaceae	*Galium*
		Richardia
		Spermacoce
	Santalaceae	*Comandra*
	Solanaceae	*Capsicum*
	Violaceae	*Viola*
**Grasses**	Poaceae	*Aegilops*
		Andropogon
		Aristida
		Bouteloua
		Bromus
		Chasmanthium
		Coleataenia
		Cynodon
		Digitaria
		Eriochloa
		Hesperostipa
		Hordeum
		Muhlenbergia
		Oryza
		Paspalum
		Phalaris

		Poa
		Pseudosasa
		Puccinellia
		Saccharum
		Sacciolepis
		Setaria
		Sporobolus
		Thinopyrum
		Triticum
**Hard mast**	Fagaceae	*Quercus*
	Juglandaceae	*Carya*
**Mosses**	Ditrichaceae	*Ceratodon*
	Funariaceae	*Funaria*
	Grimmiaceae	*Grimmia*
	Sphagnaceae	*Sphagnum*
**Sedges**	Cyperaceae	*Carex*
		Cyperus
		Eriophorum
		Fuirena
		Rhynchospora
**Shrubs**	Anacardiaceae	*Toxicodendron*
	Aquifoliaceae	*Ilex*
	Asteraceae	*Artemisia*
	Berberidaceae	*Berberis*
	Caprifoliaceae	*Symphoricarpos*
	Chloranthaceae	*Hedyosmum*
	Convolvulaceae	*Ipomoea*
	Cornaceae	*Cornus*
	Ephedraceae	*Ephedra*
	Fabaceae	*Acacia*
		Calliandra
		Prosopis
		Sesbania
	Hydrangeaceae	*Hydrangea*
	Lauraceae	*Persea*
	Magnoliaceae	*Magnolia*
	Moraceae	*Ficus*
	Myricaceae	*Morella*
	Rhamnaceae	*Ceanothus*
		Frangula

	Rosaceae	*Coleogyne*
	Zygophyllaceae	*Larrea*
**Soft mast**	Ericaceae	*Vaccinium*
	Grossulariaceae	*Ribes*
	Moraceae	*Morus*
	Rosaceae	*Prunus*
		Rubus
	Solanaceae	*Solanum*
	Ulmaceae	*Celtis*
**Rushes**	Juncaceae	*Juncus*
		Luzula
	Sparganiaceae	*Sparganium*
	Typhaceae	*Typha*
**Woody vines**	Aristolochiaceae	*Aristolochia*
	Fabaceae	*Wisteria*
	Smilacaceae	*Smilax*
	Vitaceae	*Parthenocissus*
		Vitis

Table S4 Results of SIMPER of monthly pairwise comparisons for plant family contribution to the diet of wild pigs (*Sus scrofa*) in South Carolina, U.S. Only plant families that contributed to at least 70% of the differences between months were included. Average = average contribution of each plant family to average dissimilarity between the two months; SD = standard deviation of contribution of each plant family; Ratio = a measure of variance (ratio of SD to average) for each plant family; Cumulative contribution = rank ordered (highest to lowest) cumulative contribution of each plant family based on average and expressed as a proportion; P = permutation p-value.

Month Comparison	Plant Family	Average	SD	Ratio	Cumulative Contribution	P
December-January	Poaceae	0.126	0.114	1.103	0.251	0.900
December-January	Fagaceae	0.104	0.080	1.306	0.460	0.260
December-January	Fabaceae	0.082	0.064	1.270	0.623	0.441
December-January	Rosaceae	0.073	0.112	0.648	0.768	0.595
December-February	Rosaceae	0.149	0.176	0.851	0.260	0.001
December-February	Poaceae	0.122	0.078	1.560	0.472	0.937

December-February	Fagaceae	0.103	0.089	1.159	0.651	0.277
December-February	Fabaceae	0.090	0.070	1.293	0.807	0.271
December-March	Poaceae	0.141	0.093	1.520	0.239	0.598
December-March	Pinaceae	0.123	0.109	1.135	0.448	0.001
December-March	Fabaceae	0.101	0.075	1.346	0.620	0.067
December-March	Fagaceae	0.099	0.078	1.271	0.789	0.357
December-April	Poaceae	0.145	0.093	1.559	0.229	0.479
December-April	Smilacaceae	0.119	0.129	0.924	0.417	0.001
December-April	Fagaceae	0.096	0.078	1.230	0.568	0.443
December-April	Fabaceae	0.070	0.052	1.350	0.679	0.759
December-April	Pinaceae	0.060	0.068	0.885	0.774	0.258
December-May	Rosaceae	0.168	0.217	0.776	0.247	0.001
December-May	Poaceae	0.124	0.073	1.707	0.429	0.882
December-May	Fagaceae	0.097	0.075	1.289	0.572	0.398
December-May	Fabaceae	0.068	0.051	1.326	0.671	0.763
December-May	Vitaceae	0.067	0.190	0.350	0.769	0.044
December-June	Poaceae	0.140	0.091	1.533	0.219	0.618
December-June	Fagaceae	0.111	0.082	1.354	0.392	0.200
December-June	Fabaceae	0.072	0.055	1.327	0.505	0.707
December-June	Pinaceae	0.059	0.080	0.739	0.597	0.258
December-June	Vitaceae	0.049	0.092	0.530	0.673	0.108
December-June	Rosaceae	0.048	0.074	0.641	0.747	0.911
December-July	Fagaceae	0.141	0.146	0.962	0.213	0.010
December-July	Poaceae	0.138	0.111	1.250	0.422	0.670
December-July	Fabaceae	0.098	0.088	1.115	0.570	0.108
December-July	Rosaceae	0.080	0.112	0.708	0.691	0.444
December-July	Polygonaceae	0.063	0.108	0.580	0.785	0.017
December-August	Poaceae	0.118	0.083	1.420	0.204	0.969
December-August	Fagaceae	0.099	0.078	1.270	0.376	0.360
December-August	Fabaceae	0.070	0.051	1.377	0.498	0.762
December-August	Vitaceae	0.059	0.104	0.566	0.600	0.051
December-August	Polygonaceae	0.055	0.064	0.863	0.696	0.049
December-August	Pinaceae	0.052	0.063	0.815	0.785	0.409
December-September	Poaceae	0.118	0.080	1.487	0.177	0.971
December-September	Fagaceae	0.102	0.094	1.091	0.329	0.299
December-September	Arecaceae	0.092	0.186	0.494	0.467	0.001
December-September	Fabaceae	0.072	0.057	1.259	0.575	0.741
December-September	Pinaceae	0.044	0.067	0.662	0.641	0.656
December-September	Juglandaceae	0.039	0.089	0.435	0.698	0.162
December-September	Rosaceae	0.037	0.067	0.548	0.753	0.979
December-October	Poaceae	0.131	0.091	1.437	0.243	0.813
December-October	Fagaceae	0.114	0.108	1.058	0.453	0.130
December-October	Juglandaceae	0.083	0.159	0.521	0.607	0.002
December-October	Fabaceae	0.063	0.047	1.345	0.723	0.925

December-November	Poaceae	0.145	0.115	1.256	0.272	0.473
December-November	Fagaceae	0.108	0.088	1.231	0.474	0.240
December-November	Fabaceae	0.083	0.105	0.788	0.630	0.409
December-November	Pinaceae	0.080	0.090	0.888	0.780	0.036
January-February	Rosaceae	0.149	0.156	0.950	0.269	0.003
January-February	Poaceae	0.140	0.106	1.314	0.521	0.615
January-February	Fabaceae	0.096	0.072	1.328	0.695	0.119
January-February	Fagaceae	0.078	0.102	0.766	0.836	0.800
January-March	Poaceae	0.168	0.124	1.349	0.274	0.057
January-March	Pinaceae	0.119	0.111	1.075	0.469	0.001
January-March	Fabaceae	0.108	0.080	1.352	0.645	0.017
January-March	Rosaceae	0.077	0.099	0.775	0.770	0.504
January-April	Poaceae	0.185	0.142	1.309	0.281	0.010
January-April	Smilacaceae	0.112	0.122	0.921	0.451	0.001
January-April	Fabaceae	0.083	0.068	1.219	0.577	0.400
January-April	Rosaceae	0.082	0.108	0.764	0.702	0.441
January-May	Rosaceae	0.178	0.186	0.954	0.277	0.001
January-May	Poaceae	0.155	0.112	1.385	0.518	0.270
January-May	Fabaceae	0.078	0.062	1.263	0.640	0.524
January-May	Vitaceae	0.064	0.185	0.349	0.740	0.055
January-June	Poaceae	0.180	0.137	1.310	0.272	0.016
January-June	Rosaceae	0.087	0.109	0.801	0.405	0.349
January-June	Fabaceae	0.084	0.076	1.098	0.532	0.359
January-June	Fagaceae	0.078	0.088	0.883	0.650	0.777
January-June	Polygonaceae	0.048	0.068	0.713	0.723	0.143
January-July	Poaceae	0.178	0.151	1.179	0.277	0.013
January-July	Rosaceae	0.106	0.123	0.865	0.442	0.105
January-July	Fabaceae	0.105	0.097	1.084	0.606	0.033
January-July	Fagaceae	0.090	0.156	0.580	0.747	0.539
January-August	Poaceae	0.136	0.109	1.249	0.228	0.698
January-August	Fabaceae	0.078	0.073	1.062	0.358	0.551
January-August	Fagaceae	0.076	0.086	0.891	0.486	0.796
January-August	Rosaceae	0.065	0.100	0.652	0.595	0.723
January-August	Polygonaceae	0.059	0.066	0.891	0.694	0.022
January-August	Vitaceae	0.057	0.101	0.565	0.789	0.056
January-September	Poaceae	0.153	0.121	1.268	0.218	0.277
January-September	Fagaceae	0.090	0.106	0.856	0.347	0.532
January-September	Arecaceae	0.089	0.182	0.488	0.474	0.001
January-September	Fabaceae	0.079	0.073	1.085	0.586	0.521
January-September	Rosaceae	0.074	0.101	0.733	0.691	0.585
January-September	Juglandaceae	0.038	0.086	0.442	0.746	0.163
January-October	Poaceae	0.138	0.102	1.343	0.233	0.686
January-October	Fagaceae	0.131	0.113	1.157	0.456	0.030
January-October	Juglandaceae	0.081	0.154	0.524	0.593	0.003

January-October	Fabaceae	0.070	0.067	1.043	0.711	0.807
January-November	Poaceae	0.150	0.119	1.262	0.262	0.350
January-November	Fagaceae	0.110	0.102	1.073	0.453	0.210
January-November	Fabaceae	0.090	0.107	0.842	0.610	0.254
January-November	Pinaceae	0.065	0.090	0.721	0.724	0.129
February-March	Poaceae	0.145	0.100	1.447	0.232	0.463
February-March	Rosaceae	0.138	0.156	0.884	0.452	0.008
February-March	Pinaceae	0.108	0.096	1.120	0.624	0.001
February-March	Fabaceae	0.099	0.076	1.316	0.783	0.082
February-April	Rosaceae	0.148	0.167	0.885	0.214	0.005
February-April	Poaceae	0.147	0.110	1.345	0.427	0.417
February-April	Smilacaceae	0.100	0.109	0.913	0.571	0.001
February-April	Fabaceae	0.094	0.076	1.239	0.707	0.192
February-May	Rosaceae	0.191	0.170	1.126	0.295	0.003
February-May	Poaceae	0.130	0.090	1.454	0.495	0.744
February-May	Fabaceae	0.086	0.063	1.368	0.628	0.376
February-May	Vitaceae	0.059	0.169	0.348	0.719	0.083
February-June	Rosaceae	0.150	0.166	0.904	0.212	0.001
February-June	Poaceae	0.149	0.103	1.441	0.423	0.371
February-June	Fabaceae	0.106	0.085	1.237	0.573	0.037
February-June	Fagaceae	0.084	0.104	0.807	0.691	0.675
February-June	Pinaceae	0.042	0.074	0.571	0.751	0.709
February-July	Rosaceae	0.157	0.163	0.966	0.231	0.003
February-July	Poaceae	0.149	0.112	1.325	0.450	0.368
February-July	Fabaceae	0.114	0.091	1.250	0.618	0.007
February-July	Fagaceae	0.099	0.159	0.623	0.763	0.341
February-August	Rosaceae	0.135	0.161	0.839	0.202	0.009
February-August	Poaceae	0.126	0.090	1.409	0.390	0.882
February-August	Fabaceae	0.099	0.080	1.235	0.538	0.087
February-August	Fagaceae	0.082	0.097	0.841	0.660	0.708
February-August	Vitaceae	0.051	0.090	0.565	0.736	0.108
February-September	Rosaceae	0.130	0.152	0.858	0.178	0.013
February-September	Poaceae	0.129	0.091	1.427	0.355	0.864
February-September	Fabaceae	0.092	0.076	1.210	0.480	0.201
February-September	Fagaceae	0.092	0.106	0.867	0.606	0.529
February-September	Arecaceae	0.081	0.167	0.487	0.717	0.001
February-October	Poaceae	0.130	0.095	1.366	0.199	0.836
February-October	Fagaceae	0.124	0.108	1.147	0.389	0.061
February-October	Rosaceae	0.124	0.148	0.834	0.579	0.028
February-October	Fabaceae	0.091	0.074	1.233	0.718	0.191
February-November	Poaceae	0.145	0.108	1.343	0.236	0.465
February-November	Rosaceae	0.118	0.138	0.852	0.427	0.046
February-November	Fagaceae	0.106	0.100	1.062	0.599	0.257
February-November	Fabaceae	0.097	0.095	1.030	0.757	0.140

March-April	Poaceae	0.141	0.127	1.110	0.205	0.558
March-April	Pinaceae	0.117	0.108	1.086	0.376	0.001
March-April	Smilacaceae	0.109	0.120	0.903	0.535	0.001
March-April	Fabaceae	0.105	0.081	1.303	0.688	0.051
March-April	Rosaceae	0.051	0.060	0.849	0.763	0.871
March-May	Rosaceae	0.163	0.188	0.865	0.228	0.003
March-May	Poaceae	0.139	0.102	1.364	0.422	0.606
March-May	Pinaceae	0.113	0.105	1.072	0.580	0.002
March-May	Fabaceae	0.097	0.070	1.383	0.715	0.148
March-June	Poaceae	0.147	0.123	1.197	0.203	0.411
March-June	Pinaceae	0.126	0.115	1.094	0.377	0.001
March-June	Fabaceae	0.117	0.088	1.339	0.539	0.006
March-June	Rosaceae	0.058	0.069	0.839	0.618	0.825
March-June	Fagaceae	0.054	0.074	0.729	0.693	0.981
March-June	Vitaceae	0.045	0.086	0.524	0.755	0.135
March-July	Poaceae	0.148	0.131	1.125	0.207	0.402
March-July	Fabaceae	0.127	0.099	1.289	0.385	0.001
March-July	Pinaceae	0.126	0.122	1.038	0.562	0.001
March-July	Rosaceae	0.081	0.100	0.817	0.676	0.441
March-July	Fagaceae	0.059	0.147	0.402	0.759	0.971
March-August	Poaceae	0.154	0.100	1.544	0.221	0.252
March-August	Fabaceae	0.110	0.083	1.324	0.378	0.015
March-August	Pinaceae	0.109	0.100	1.093	0.534	0.001
March-August	Fagaceae	0.058	0.077	0.755	0.618	0.976
March-August	Vitaceae	0.055	0.098	0.561	0.697	0.058
March-August	Polygonaceae	0.050	0.060	0.847	0.769	0.109
March-September	Poaceae	0.126	0.107	1.182	0.165	0.902
March-September	Pinaceae	0.106	0.104	1.016	0.303	0.001
March-September	Fabaceae	0.103	0.081	1.266	0.437	0.048
March-September	Arecaceae	0.086	0.178	0.485	0.550	0.001
March-September	Fagaceae	0.078	0.105	0.741	0.652	0.775
March-September	Rosaceae	0.049	0.064	0.755	0.716	0.935
March-October	Poaceae	0.162	0.108	1.491	0.226	0.127
March-October	Fagaceae	0.132	0.116	1.142	0.411	0.017
March-October	Fabaceae	0.101	0.077	1.310	0.552	0.072
March-October	Pinaceae	0.099	0.093	1.061	0.691	0.001
March-October	Juglandaceae	0.078	0.152	0.516	0.801	0.002
March-November	Poaceae	0.172	0.124	1.386	0.272	0.045
March-November	Fabaceae	0.107	0.103	1.042	0.441	0.050
March-November	Fagaceae	0.104	0.107	0.980	0.606	0.284
March-November	Pinaceae	0.103	0.096	1.077	0.769	0.001
April-May	Rosaceae	0.176	0.203	0.865	0.241	0.002
April-May	Poaceae	0.125	0.099	1.258	0.413	0.836
April-May	Smilacaceae	0.100	0.108	0.932	0.551	0.001

April-May	Fabaceae	0.069	0.054	1.295	0.647	0.702
April-May	Vitaceae	0.067	0.192	0.351	0.739	0.072
April-June	Poaceae	0.124	0.105	1.183	0.176	0.876
April-June	Smilacaceae	0.119	0.126	0.946	0.345	0.001
April-June	Fagaceae	0.072	0.073	0.991	0.448	0.811
April-June	Fabaceae	0.063	0.050	1.258	0.537	0.874
April-June	Rosaceae	0.062	0.077	0.804	0.626	0.732
April-June	Vitaceae	0.050	0.094	0.532	0.696	0.117
April-June	Polygonaceae	0.047	0.062	0.764	0.763	0.211
April-July	Poaceae	0.126	0.114	1.112	0.174	0.855
April-July	Smilacaceae	0.119	0.133	0.893	0.338	0.001
April-July	Fabaceae	0.093	0.092	1.010	0.466	0.212
April-July	Rosaceae	0.089	0.108	0.825	0.589	0.330
April-July	Fagaceae	0.084	0.150	0.559	0.704	0.638
April-August	Poaceae	0.164	0.113	1.459	0.230	0.119
April-August	Smilacaceae	0.109	0.118	0.921	0.383	0.001
April-August	Fagaceae	0.068	0.078	0.869	0.477	0.876
April-August	Vitaceae	0.060	0.106	0.564	0.561	0.059
April-August	Fabaceae	0.059	0.049	1.200	0.643	0.916
April-August	Polygonaceae	0.055	0.063	0.869	0.720	0.073
April-September	Poaceae	0.105	0.087	1.201	0.138	0.992
April-September	Smilacaceae	0.102	0.116	0.881	0.273	0.001
April-September	Arecaceae	0.092	0.189	0.490	0.395	0.004
April-September	Fagaceae	0.084	0.105	0.800	0.506	0.637
April-September	Fabaceae	0.066	0.060	1.101	0.593	0.828
April-September	Rosaceae	0.051	0.071	0.717	0.661	0.846
April-September	Typhaceae	0.045	0.125	0.361	0.720	0.012
April-October	Poaceae	0.182	0.127	1.436	0.254	0.019
April-October	Fagaceae	0.126	0.120	1.045	0.429	0.073
April-October	Smilacaceae	0.097	0.108	0.901	0.565	0.001
April-October	Juglandaceae	0.085	0.160	0.532	0.683	0.004
April-October	Fabaceae	0.052	0.044	1.187	0.756	0.974
April-November	Poaceae	0.193	0.141	1.373	0.280	0.008
April-November	Fagaceae	0.107	0.103	1.033	0.434	0.270
April-November	Smilacaceae	0.093	0.104	0.893	0.569	0.001
April-November	Fabaceae	0.079	0.110	0.719	0.684	0.507
April-November	Pinaceae	0.062	0.090	0.687	0.773	0.242
May-June	Rosaceae	0.181	0.200	0.906	0.246	0.001
May-June	Poaceae	0.130	0.097	1.334	0.423	0.787
May-June	Vitaceae	0.103	0.191	0.539	0.562	0.005
May-June	Fabaceae	0.069	0.061	1.139	0.656	0.723
May-June	Fagaceae	0.052	0.072	0.721	0.727	0.976
May-July	Rosaceae	0.190	0.196	0.969	0.263	0.001
May-July	Poaceae	0.131	0.108	1.209	0.445	0.769

May-July	Fabaceae	0.091	0.085	1.069	0.571	0.242
May-July	Vitaceae	0.067	0.193	0.346	0.664	0.052
May-July	Polygonaceae	0.064	0.089	0.720	0.753	0.036
May-August	Rosaceae	0.156	0.196	0.799	0.217	0.006
May-August	Poaceae	0.137	0.091	1.508	0.408	0.625
May-August	Vitaceae	0.104	0.179	0.584	0.553	0.003
May-August	Fabaceae	0.064	0.059	1.086	0.642	0.870
May-August	Fagaceae	0.057	0.076	0.754	0.721	0.950
May-September	Rosaceae	0.153	0.182	0.841	0.196	0.007
May-September	Poaceae	0.111	0.084	1.327	0.338	0.972
May-September	Arecaceae	0.085	0.174	0.488	0.447	0.015
May-September	Fagaceae	0.076	0.102	0.745	0.544	0.761
May-September	Vitaceae	0.072	0.172	0.422	0.637	0.029
May-September	Fabaceae	0.067	0.060	1.115	0.722	0.757
May-October	Poaceae	0.152	0.100	1.524	0.203	0.317
May-October	Rosaceae	0.142	0.179	0.793	0.391	0.016
May-October	Fagaceae	0.130	0.113	1.155	0.565	0.073
May-October	Juglandaceae	0.077	0.148	0.517	0.667	0.014
May-October	Vitaceae	0.058	0.166	0.349	0.744	0.084
May-November	Poaceae	0.166	0.116	1.436	0.230	0.139
May-November	Rosaceae	0.138	0.165	0.836	0.421	0.037
May-November	Fagaceae	0.102	0.104	0.980	0.563	0.343
May-November	Fabaceae	0.079	0.099	0.800	0.672	0.509
May-November	Pinaceae	0.061	0.087	0.702	0.757	0.240
June-July	Poaceae	0.134	0.120	1.122	0.188	0.717
June-July	Fagaceae	0.100	0.160	0.625	0.328	0.350
June-July	Rosaceae	0.094	0.110	0.857	0.459	0.252
June-July	Polygonaceae	0.083	0.103	0.801	0.575	0.001
June-July	Fabaceae	0.081	0.102	0.790	0.688	0.456
June-July	Vitaceae	0.050	0.096	0.518	0.758	0.103
June-August	Poaceae	0.161	0.107	1.505	0.242	0.141
June-August	Vitaceae	0.086	0.110	0.781	0.370	0.001
June-August	Fagaceae	0.083	0.086	0.962	0.495	0.639
June-August	Polygonaceae	0.064	0.066	0.972	0.591	0.013
June-August	Rosaceae	0.043	0.062	0.702	0.656	0.935
June-August	Fabaceae	0.033	0.032	1.032	0.706	1.000
June-September	Poaceae	0.112	0.091	1.236	0.152	0.986
June-September	Fagaceae	0.097	0.109	0.896	0.283	0.397
June-September	Arecaceae	0.094	0.191	0.496	0.411	0.001
June-September	Rosaceae	0.057	0.077	0.740	0.488	0.813
June-September	Vitaceae	0.054	0.088	0.616	0.561	0.070
June-September	Fabaceae	0.051	0.062	0.829	0.630	0.991
June-September	Polygonaceae	0.048	0.056	0.856	0.694	0.148
June-September	Juglandaceae	0.041	0.090	0.457	0.750	0.144

June-October	Poaceae	0.179	0.119	1.497	0.259	0.017
June-October	Fagaceae	0.136	0.118	1.161	0.457	0.017
June-October	Juglandaceae	0.086	0.162	0.530	0.582	0.001
June-October	Vitaceae	0.040	0.075	0.535	0.641	0.200
June-October	Rosaceae	0.039	0.055	0.720	0.698	0.971
June-October	Polygonaceae	0.036	0.046	0.779	0.750	0.514
June-November	Poaceae	0.189	0.136	1.390	0.278	0.009
June-November	Fagaceae	0.114	0.106	1.084	0.447	0.162
June-November	Fabaceae	0.073	0.120	0.606	0.553	0.680
June-November	Pinaceae	0.070	0.093	0.749	0.656	0.113
June-November	Polygonaceae	0.043	0.050	0.860	0.719	0.294
July-August	Poaceae	0.161	0.120	1.335	0.229	0.165
July-August	Fagaceae	0.099	0.151	0.656	0.371	0.338
July-August	Polygonaceae	0.080	0.086	0.931	0.485	0.001
July-August	Rosaceae	0.071	0.100	0.713	0.587	0.613
July-August	Fabaceae	0.071	0.096	0.741	0.689	0.743
July-August	Vitaceae	0.060	0.107	0.556	0.774	0.046
July-September	Fagaceae	0.117	0.161	0.724	0.154	0.134
July-September	Poaceae	0.112	0.101	1.108	0.301	0.989
July-September	Arecaceae	0.092	0.190	0.486	0.423	0.001
July-September	Fabaceae	0.080	0.097	0.829	0.529	0.486
July-September	Rosaceae	0.080	0.102	0.782	0.634	0.457
July-September	Polygonaceae	0.065	0.085	0.769	0.720	0.011
July-October	Poaceae	0.179	0.128	1.398	0.247	0.022
July-October	Fagaceae	0.164	0.154	1.067	0.474	0.001
July-October	Juglandaceae	0.084	0.162	0.519	0.591	0.001
July-October	Rosaceae	0.064	0.091	0.704	0.680	0.728
July-October	Fabaceae	0.063	0.087	0.718	0.766	0.915
July-November	Poaceae	0.187	0.144	1.300	0.268	0.013
July-November	Fagaceae	0.137	0.149	0.919	0.464	0.042
July-November	Fabaceae	0.094	0.126	0.750	0.599	0.163
July-November	Pinaceae	0.066	0.094	0.701	0.693	0.133
July-November	Rosaceae	0.065	0.086	0.761	0.786	0.687
August-September	Poaceae	0.138	0.094	1.478	0.196	0.650
August-September	Arecaceae	0.093	0.172	0.542	0.328	0.001
August-September	Fagaceae	0.090	0.100	0.901	0.456	0.528
August-September	Vitaceae	0.060	0.095	0.633	0.541	0.032
August-September	Polygonaceae	0.051	0.055	0.925	0.613	0.105
August-September	Fabaceae	0.043	0.060	0.714	0.674	0.998
August-September	Juglandaceae	0.036	0.085	0.431	0.726	0.201
August-October	Poaceae	0.128	0.093	1.384	0.219	0.863
August-October	Fagaceae	0.122	0.110	1.115	0.428	0.067
August-October	Juglandaceae	0.078	0.150	0.519	0.562	0.002
August-October	Vitaceae	0.049	0.088	0.563	0.646	0.107

August-October	Polygonaceae	0.042	0.050	0.850	0.718	0.280
August-November	Poaceae	0.144	0.109	1.317	0.244	0.478
August-November	Fagaceae	0.105	0.097	1.089	0.423	0.281
August-November	Fabaceae	0.066	0.112	0.590	0.535	0.816
August-November	Pinaceae	0.059	0.086	0.679	0.634	0.280
August-November	Vitaceae	0.047	0.085	0.557	0.714	0.131
September-October	Poaceae	0.156	0.105	1.488	0.226	0.224
September-October	Fagaceae	0.119	0.110	1.079	0.398	0.098
September-October	Juglandaceae	0.093	0.147	0.629	0.532	0.001
September-October	Arecaceae	0.084	0.160	0.524	0.654	0.001
September-October	Fabaceae	0.039	0.053	0.726	0.710	1.000
September-November	Poaceae	0.166	0.123	1.349	0.237	0.107
September-November	Fagaceae	0.106	0.098	1.082	0.388	0.243
September-November	Arecaceae	0.077	0.159	0.481	0.497	0.008
September-November	Fabaceae	0.072	0.106	0.674	0.600	0.693
September-November	Pinaceae	0.058	0.085	0.681	0.682	0.272
September-November	Rosaceae	0.036	0.054	0.681	0.734	0.964
October-November	Poaceae	0.134	0.099	1.351	0.243	0.709
October-November	Fagaceae	0.116	0.097	1.187	0.453	0.165
October-November	Juglandaceae	0.069	0.133	0.520	0.578	0.022
October-November	Fabaceae	0.060	0.103	0.582	0.687	0.924
October-November	Pinaceae	0.055	0.081	0.674	0.786	0.336

References

1. Oksanen, J. *et al.* vegan: Community Ecology Package. (2022).

2. Mendiburu, F. de. agricolae: Statistical Procedures for Agricultural Research. (2021).
